# A NADPH-Dependent Aldo/Keto Reductase Is Responsible for Detoxifying 3-Keto-Deoxynivalenol to 3-*epi*-Deoxynivalenol in *Pelagibacterium halotolerans* ANSP101

**DOI:** 10.3390/foods13071064

**Published:** 2024-03-29

**Authors:** Yanrong Liu, Mingxin Ma, Yu Tang, Zhenqian Huang, Yongpeng Guo, Qiugang Ma, Lihong Zhao

**Affiliations:** 1 State Key Laboratory of Animal Nutrition and Feeding, Poultry Nutrition and Feed Technology Innovation Team, College of Animal Science and Technology, China Agricultural University, Beijing 100193, China; 15110578158@163.com (Y.L.); mamingxin@cau.edu.cn (M.M.); m17801115235@163.com (Y.T.); hzq0921@cau.edu.cn (Z.H.); maqiugang@cau.edu.cn (Q.M.); 2 College of Animal Science and Technology, Henan Agricultural University, Zhengzhou 450046, China; guoyp@henau.edu.cn

**Keywords:** Deoxynivalenol (DON), 3-keto-DON, 3-*epi*-DON, aldo/keto reductase, biodegradation

## Abstract

Deoxynivalenol (DON), primarily generated by *Fusarium* species, often exists in agricultural products. It can be transformed to 3-*epi*-deoxynivalenol (3-*epi*-DON), with a relatively low toxicity, via two steps. DDH in *Pelagibacterium halotolerans* ANSP101 was proved to convert DON to 3-keto-deoxynivalenol (3-keto-DON). In the present research, AKR4, a NADPH-dependent aldo/keto reductase from *P. halotolerans* ANSP101, was identified to be capable of converting 3-keto-DON into 3-*epi*-DON. Our results demonstrated that AKR4 is clearly a NADPH-dependent enzyme, for its utilization of NADPH is higher than that of NADH. AKR4 functions at a range of pH 5–10 and temperatures of 20–60 °C. AKR4 is able to degrade 89% of 3-keto-DON in 90 min at pH 7 and 50 °C with NADPH as the cofactor. The discovery of AKR4, serving as an enzyme involved in the final step in DON degradation, might provide an option for the final detoxification of DON in food and feed.

## 1. Introduction

Mycotoxins are produced primarily by *Penicillium*, *Aspergillus*, and *Fusarium* [1]. They widely contaminate agricultural products, cause serious harm to the health of humans and livestock, and cause economic losses to agricultural and food industries. According to the data from the Food and Agriculture Organization (FAO), more than 25% of crops worldwide were contaminated with mycotoxins [2]. Further research showed that the FAO’s previous estimates of mycotoxin contamination had been underestimated [3]. In addition, mycotoxins are difficult to eliminate during processing because of their structural strength [4].

Deoxynivalenol (DON) is a mycotoxin mainly produced by *Fusarium* [5]. DON can cause emesis and loss of appetite when ingested by animals [6], whereas the long-term ingestion of DON causes animals to experience chronic intestinal bowel diseases, immune inhibition, susceptibility to infections, and reduced growth performance and reproductive performance [7,8]. Because of its toxicity and ubiquity, DON is considered a serious concern for food and feed security [9]. To minimize DON contamination, a large number of detoxification methods have been tested. However, few physical and chemical methods can be used to remove DON effectively and economically. For example, inert adsorbents are widely used to adsorb mycotoxins in feed industry [10]. Nevertheless, binding of mycotoxins to adsorbents is only effective against a limited variety of mycotoxins, such as AFB_1_, and has little effect on DON. Biological degradation is an effective method of DON detoxification which has garnered significant interest in recent years.

It has been reported that DON is able to be degraded by some bacteria and enzymes. The de-epoxidation of DON occurs in several aerobic or anaerobic microorganisms [11,12,13]. It has been proved that DON can be hydroxylated to 16-HDON by the *Sphingomonas* strain KSM1 [14]. The glycosylation, acetylation, or glutathionylation of DON usually occurs in plants to reduce toxicity [15,16]. However, masked mycotoxins produced during digestion can be hydrolyzed to DON [17]. The oxidation and subsequent reduction of the C3 position of DON are also promising detoxification methods. The toxicity of both 3-keto-DON and 3-*epi*-DON are lower than DON, especially 3-*epi*-DON [18]. To date, several bacterial isolates which convert DON to 3-*epi*-DON have been reported, such as *Devosia mutans* 17-2-E-8 [19], *Sphingomonas* sp. S3-4 [20], and *Devosia* sp. D6-9 [21]. Furthermore, several enzymes participate in the oxidation or reduction of the C3 position. Most reported enzymes involved in this pathway include dehydrogenase and aldo/keto reductase. Enzymes which convert DON to 3-keto-DON include DepA in *D. mutans* 17-2-E-8 [22], AKR18A1 in *Sphingomonas* sp. S3-4 [20], and QDDH in *Devosia* sp. D6-9 [21]. The enzymes which convert 3-keto-DON to 3-*epi*-DON include DepB from *D. mutans* 17-2-E-8 [23], and two NADPH-dependent reductases, AKR13B2 and AKR6D1, from *Devosia* sp. D6-9 [21].

**Figure 1:**
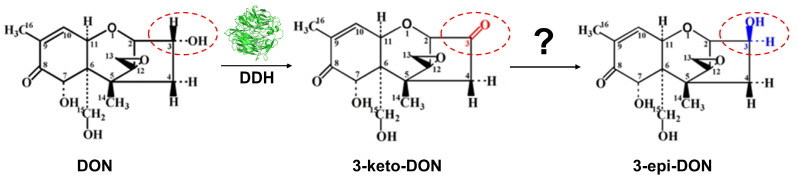
Transformation of Deoxynivalenol (DON) to 3-*epi*-DON. The reaction involves oxidation of the DON C3 hydroxyl group followed by reduction of the 3-keto-DON C3 ketone.

In previous studies, we screened a new bacterium, *Pelagibacterium halotolerans* ANSP101, capable of degrading DON, and characterized a dehydrogenase DDH which oxidized DON to 3-keto-DON [24,25] (Figure 1). The aim of our research is to isolate the second enzyme in DON epimerization from this strain. We successfully identified AKR4, which is capable of reducing 3-keto-DON to 3-*epi*-DON in *P. halotolerans* ANSP101. Using enzymes to reduce DON levels in food and feed will be an important post-harvest strategy for protecting the health of human and animals.

## 2. Materials and Methods

### 2.1. Chemiclas and Reagents

The 3-keto-DON (purity ≥ 98%) was from TripleBond (Guelph, ON, Canada). The standards were made with 5 mg/mL stock solution in acetonitrile solvent. Restriction enzymes and T4 DNA lignase were ordered from New England Biolabs (Beijing, China). The 2 × Taq plus PCR MasterMix, DL2000 DNA marker, DNA loading buffer, and GelRed were purchased from Aidlab Co., Ltd. (Beijing, China). The Ni-NTA column was purchased from TransGen Biotech Co., Ltd. (Shanghai, China). The other chemicals were from Sinopharm Chemical Reagent Co., Ltd. (Beijing, China).

### 2.2. 3-Keto-DON Detection by HPLC

A Shimadzu LC-10 AT high-performance liquid chromatography system (Shimadzu, Tokyo, Japan) was used to detect 3-keto-DON. A reversed phase C18 column (4.6 mm × 250 mm, 5 μm) was used to separate the substances. The mobile phase of 3-keto-DON was 23% of acetonitrile. The injection volume was 20 µL. The detection wavelength of 3-keto-DON was 218 nm. The peak time of 3-keto-DON was 5.79 min. The flow rate of the mobile phase was 1 mL/min.

### 2.3. 3-Keto-DON and 3-epi-DON Identification by HPLC-MS/MS

A sample containing 60 µg/mL 3-keto-DON, 100 µg/mL AKR4, and 1 mM NADPH was cultured for 24 h at 50 °C. After incubation, the sample was subjected to HPLC to confirm the 3-keto-DON was degraded completely. The remaining sample was blown dry and solubilized with 200 µL methanol, filtered through a 0.22 µm filter membrane, and subjected to HPLC-MS/MS analysis in the positive mode. The 3-keto-DON standards and the degradation products of 3-epi-DON were determined with a Waters Acquity high-performance liquid chromatography system (Waters, Milford, MA, USA) coupled to a Q Exactive Focus Orbitrap LC-MS/MS System (Thermo Fisher Scientific, Norristown, PA, USA). A C18 column (Waters HSS T3, 100 mm × 2.1 mm, 1.8 µm) was used for chromatographic separation. Sample injection volume was 1 µL. Solvent A for LC was 0.1% fomic acid and solvent B was acetonitrile. The mobile phase gradient was as follows: under initial conditions, 5% B was increased to 95% B in 15 min, then the column was washed at 95% B, and the column was re-equilibrated in 10 min. The flow rate was maintained at 0.3 mL/min. All data were analyzed with Xcalibur software-v4.1.50 (Thermo Fisher Scientific, Norristown, PA, USA).

### 2.4. Sequencing and Annotation of P. halotolerans ANSP101

The data of the whole-genome sequencing of *P. halotolerans* ANSP101 were from our previous study [25]. In brief, the bacterial DNA was isolated, and the genome was sequenced by Genewiz (Beijing, China). The functional annotation of genes was done using BLAST (Version 1.7.31+) [26]. To align the protein sequence identity between four aldo/keto reductases from *P. halotolerans* ANSP101 and DepB in *D. mutans* 17-2-E-8, BLAST analysis in the NCBI database was performed.

### 2.5. Cloning and Heterologous Expression of Recombinant Protein in E. coli

The four genes of aldo/keto reductases were amplified from the genomic DNA of *P. halotolerans* ANSP101 with the primers listed in Table S1. Both the PCR products and linearized pET-31b were treated with restriction enzymes *Nde*I and *Xho*I. The purified PCR products were cloned into the pET-31b vector and transformed into *E. coli* BL21. Recombinant proteins were expressed and purified by affinity-chromatography as previously described [23]. The purified enzymes were analyzed by SDS-PAGE.

### 2.6. Testing the Candidates for 3-Keto-DON Degradation Activity

In vitro assays to test the candidates for activity of 3-keto-DON’s transformation into 3-*epi*-DON were performed in 2 mL Eppendorf tubes. The reaction mixture contained 300 µg purified protein from each candidate suspended in 50 mM Tris-HCl buffer, pH 8, containing 500 µM NADP/NADPH and 30 µg/mL 3-keto-DON. The reaction was incubated at 30 °C for 24 h. Then, the mixture was added to an equal volume of methanol and filtered through a 0.22 µm filter before analysis by HPLC. The degradation rate of 3-keto-DON was calculated with the following formula: (1−3-keto-DON peak area in treatment/3-keto-DON peak area in control) × 100%. All assays were done at least in triplicate.

### 2.7. Degradation Characteristics of AKR4 on 3-Keto-DON

#### 2.7.1. Cofactor Specificity

A cofactor specificity assay was performed at 30 °C using 28.8 µg/mL AKR4, 500 µM NADP/NADPH, and 30 µg/mL 3-keto-DON in 50 mM Tris-HCl buffer for pH 8. The enzyme activity was measured at 30 min, 90 min, 150 min, and 24 h, respectively. All reactions were carried out in triplicate.

#### 2.7.2. Effects of Mycotoxin and Enzyme Concentration on the Removal Rate of 3-Keto-DON by AKR4

Reactions assessing the degradation effect of AKR4 on different concentrations of 3-keto-DON contained 28.8 µg/mL AKR4, 500 µM NADPH, and various concentrations of 3-keto-DON (10, 20, 30, 40, 50, and 60 µg/mL) in 50 mM Tris-HCl buffer for pH 8. Each reaction proceeded for 90 min before being stopped with methanol. The effect of different concentrations of AKR4 on degradation of 3-keto-DON was tested under the following conditions: reactions contain 50 mM Tris-HCl buffer (pH 8), 500 µM NADPH, 30 µg/mL 3-keto-DON, and various concentrations of AKR4 (3.6, 7.2, 14.4, 28.8, 43.2, and 57.6 µg/mL). The reaction was allowed to proceed for 90 min. All reactions were carried out in triplicate.

#### 2.7.3. Effects of pH and Temperature on the Activity of AKR4

The activity of AKR4 at different pH values and temperatures was examined. When examining the optimum pH of AKR4 to degrade 3-keto-DON, reactions contained 50 mM buffers for various pH (sodium citrate buffer for pH 4–5, Tris-HCl buffer for pH 6–8, and glycine-NaOH buffer for pH 9–10), 28.8 µg/mL purified AKR4, 500 µM NADPH, and 30 µg/mL 3-keto-DON, and the reaction proceeded for 90 min at 30 °C before being stopped. For incubation temperature, the mixture containing 28.8 µg/mL purified AKR4, 500 µM NADPH, and 30 µg/mL 3-keto-DON was incubated at 20–70 °C and pH 8 for 90 min. All experiments were replicated in triplicate.

#### 2.7.4. pH Stability and Thermostability of AKR4

For pH stability, AKR4 was incubated at different pH values (pH 3–9) at 4 °C for 12 or 24 h. The remaining AKR4 activity was determined at 30 °C and pH 8 with 3-keto-DON as substrate. For thermostability, AKR4 was treated at 25–50 °C and pH 8 for 0.5–2 h. Afterwards, 500 µM NADPH and 30 µg/mL 3-keto-DON were added. The mixtures were cultured at 30 °C and pH 8 for 90 min. All assays were performed in triplicate.

### 2.8. Bioinformatics Analysis

The multiple sequence alignments were carried out by the PROMALS3D multiple sequence and structure alignment server (prodata.swmed.edu/promals3d/, accessed on 5 October 2023). The homology modeling was performed by the Swiss Model server (https://swissmodel.expasy.org/interactive/, accessed on 5 October 2023). The structure of 3-keto-DON in this study was constructed in Chem3D 22.0.0. Software AutoDockTools 1.5.6 was employed for docking 3-keto-DON into the structure of AKR4. The interaction and binding affinity of the protein with a ligand complex were analyzed using Maestro 11.8.

## 3. Results

### 3.1. Identification of Aldo/Keto Reductase Genes by Microbial Genomic Sequence Analysis

**Figure 2:**
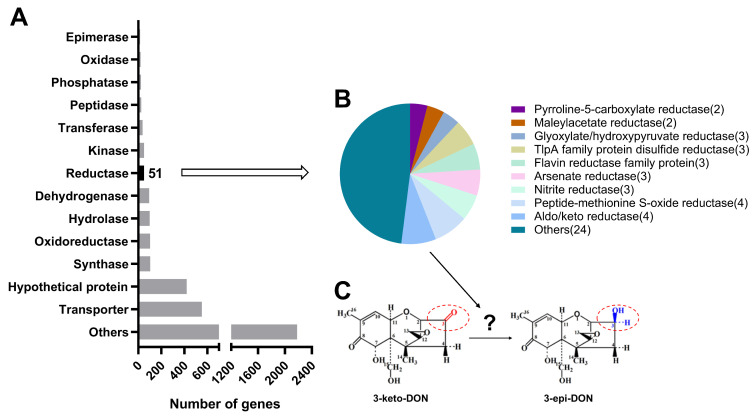
Candidate gene categories for 3-keto-DON reduction revealed by genome analysis of ANSP101. (**A**) The identification of 3-keto-DON degrading proteins was based on functional annotations and mycotoxin degradation assays. A total of 3806 protein-encoding genes were annotated, from which 51 protein sequences were filtered using the keyword “reductase”. (**B**) Four aldo/keto reductases potentially involved in the degradation of 3-keto-DON were selected. (**C**) The catalytic process from 3-keto-DON to 3-*epi*-DON.

It has been reported that DepB in *D. mutans* 17-2-E-8 can convert 3-keto-DON to 3-*epi*-DON, which is the product of a reduction of the carbonyl group at the C3 position of 3-keto-DON to hydroxyl [23]. We speculated that one or more of the aldo/keto reductases in *P. halotolerans* ANSP101 can transform 3-keto-DON to 3-*epi*-DON, and as such the *P. halotolerans* ANSP101 genome was sequenced to identify the genes of reductases. The total genome length is 3,962,298 bp. There were 3887 predicted genes, including 3806 protein-coding genes, 9 rRNA genes, and 49 tRNA genes. From the annotated protein sequences, 51 sequences were identified with a keyword filter of “reductase” (Figure 2A). The 51 sequences were further classified according to their annotations, resulting in the identification of four aldo/keto reductases (Figure 2B,C). Subsequently, the BLAST analysis was conducted to align each protein sequence’s identity between four aldo/keto reductases mentioned above and DepB reported in *D. mutans* 17-E-2-8. These four proteins showed more than 30% similarity with DepB, with AKR1 displaying the highest similarity of 44.05% (Table S2). Finally, these four proteins were selected as candidates for further validation.

### 3.2. Expression and Purification of Candidate Aldo/Keto Reductases

Genes encoding putative 3-keto-DON-degrading enzymes were amplified from *P. halotolerans* ANSP101 genomic DNA and connected to the *E. coli* expression plasmid pET31b. Four C-terminal His-tagged candidate aldo/keto reductases were successfully purified by Ni-NTA chromatography. The result of SDS-PAGE indicated that the apparent molecular weights of the four candidates were close to the predicted molecular weights of 38 kDa for AKR1, 36 kDa for AKR2, 38 kDa for AKR3, and 31 kDa for AKR4, respectively (Figure S1).

### 3.3. Degradation of 3-Keto-DON by Candidate Aldo/Keto Reductases

The 3-keto-DON degradation rates of the candidates AKR1, AKR2, AKR3, and AKR4 were determined by HPLC. The abilities of the four candidates to degrade 3-keto-DON were examined with NAD^+^, NADP^+^, NADH, and NADPH as cofactors. The assays of 3-keto-DON degradation activity of candidates showed that only AKR4 had the ability of 3-keto-DON detoxification in the presence of NADH or NADPH. However, even after incubation for 24 h, no 3-keto-DON could be degraded by AKR1, AKR2, or AKR3 with any kind of cofactor. These results suggested that AKR4 is involved in the degradation of 3-keto-DON in *P. halotolerans* ANSP101.

### 3.4. Characteristic of AKR4

**Figure 3:**
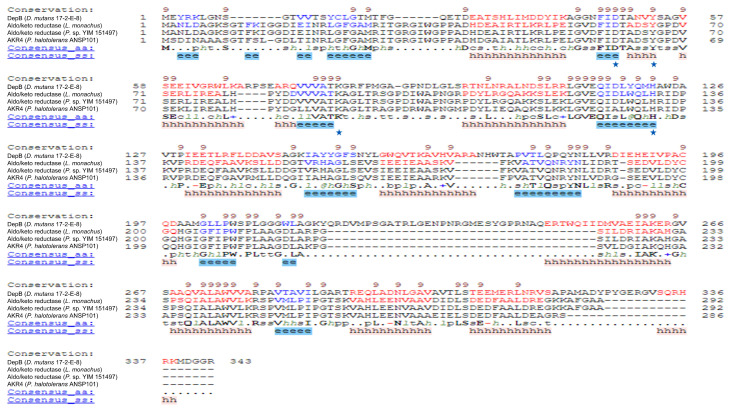
Multiple sequence alignment of AKR4 from *Pelagibacterium halotolerans* ANSP101 using the PROMALS3D multiple sequence and structure alignment server (prodata.swmed.edu/promals3d/, accessed on 5 October 2023). The α-helix was marked with red fonts, and β-strand was marked with blue fonts. The catalytic tetrad residues were marked with blue stars.

The NCBI BLAST search results showed that AKR4 displays high similarity to other aldo/keto reductases. Then the protein sequences of AKR4 (QJR19719.1), aldo/keto reductase from *Labrys monachus* (WP_307432409.1), aldo/keto reductase from *Pelagibacterium* sp. YIM 151497 (WP_264225378.1), and DepB from *D. mutans* 17-2-E-8 (KFL28068.1) were submitted to align (Figure 3). The multiple sequence alignment analysis demonstrated that all of the four proteins have the conserved catalytic tetrad of aldo/keto reductases which works as an acid-base catalyst [27].

### 3.5. Cofactor Specificity of AKR4

**Figure 4:**
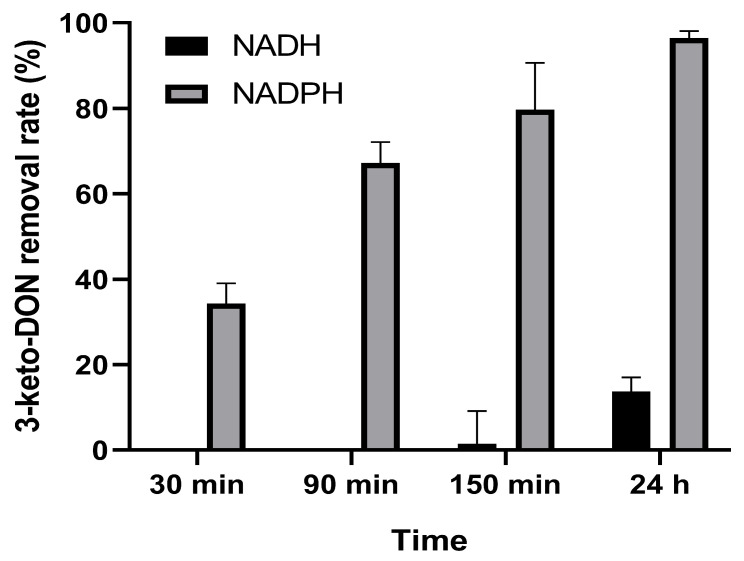
AKR4 activity using different nicotinamide cofactors. Reactions contain 50 mM of Tris-HCl buffer (pH 8), 30 µg/mL 3-keto-DON, 500 µM NADPH or NADH, and 28.8 µg/mL AKR4, and the reaction was allowed to proceed for 30 min, 90 min, 150 min, or 24 h before it was stopped with methanol.

As shown in Figure 4, the ability of AKR4 to degrade 3-keto-DON was evaluated with cofactors NADH and NADPH. The detoxification ability of AKR4 on 3-keto-DON with NADPH as a cofactor was stronger than that using NADH as a cofactor. In total, 79.75% of the 3-keto-DON was degraded by AKR4 with NADPH as a cofactor within 150 min. However, AKR4 with NADH as a cofactor was only able to degrade 1.47% of the 3-keto-DON in the same time, and was only able to degrade 13.73% of 3-keto-DON within 24 h.

### 3.6. Effects of Mycotoxin and Enzyme Concentration on the Removal Rate of 3-Keto-DON by AKR4

**Figure 5:**
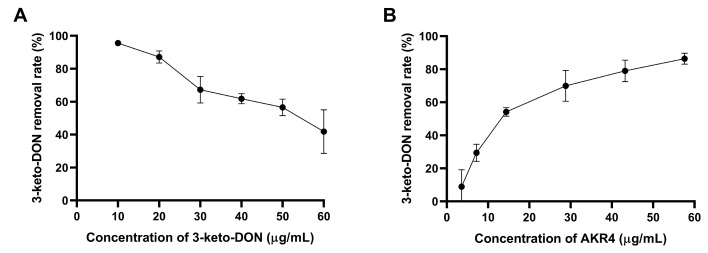
Effects of initial concentration of 3-keto-DON (**A**) and AKR4 (**B**) on the degradation of 3-keto-DON by AKR4. (**A**) Reactions contained 50 mM of Tris-HCl buffer (pH 8), 28.8 µg/mL AKR4, 500 µM NADPH, and different concentrations of 3-keto-DON (10, 20, 30, 40, 50, and 60 µg/mL). (**B**) Reactions contained 50 mM of Tris-HCl buffer (pH 8), 30 µg/mL 3-keto-DON, 500 µM NADPH, and different concentrations of AKR4 (3.6, 7.2, 14.4, 28.8, 43.2, and 57.6 µg/mL).

The effects of the concentrations of mycotoxin and enzyme on the removal rate of 3-keto-DON by AKR4 are shown in Figure 5. When the concentrations of 3-keto-DON were 10 µg/mL and 20 µg/mL, the removal rate reached more than 80% in 90 min (Figure 5A). As the concentration of AKR4 increased, the removal rate of 3-keto-DON gradually increased. A 57.6 µg/mL dose of AKR4 could degrade 86.36% of the 3-keto-DON in 90 min (Figure 5B).

### 3.7. Effects of pH and Temperature on the Removal Rate of 3-Keto-DON by AKR4

**Figure 6:**
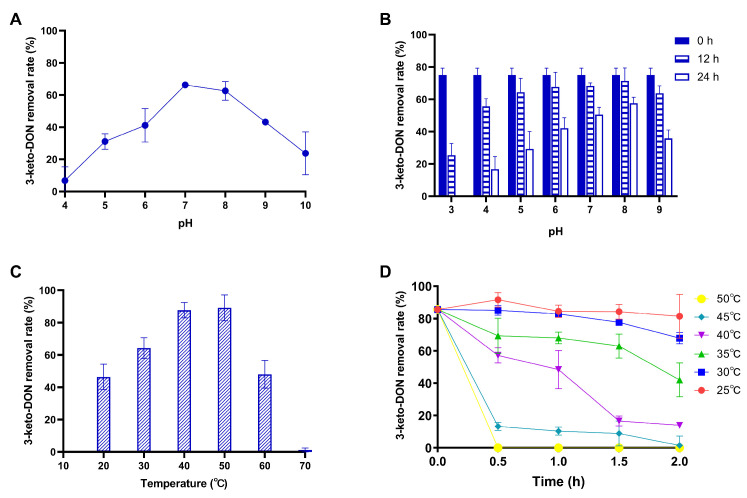
Effects of pH and temperature on the degradation of 3-keto-DON by AKR4. (**A**) The effect of pH on the activity of AKR4 was measured at 30 °C using 3-keto-DON as substrate and was performed at different pH values (pH 4–10). (**B**) The purified AKR4 was measured for residual activity after incubating at 4 °C and different pH values (pH 3–9) for 12 or 24 h before. The residual activity was calculated at 30 °C and pH 8 using 3-keto-DON as substrate. (**C**) The effect of temperature on the activity of AKR4 with 3-keto-DON as substrate was measured at different temperatures (20–70 °C) and pH 8 for 90 min. (**D**) The purified AKR4 was measured for residual activity after incubating at six elevated temperatures (25–50 °C) for 2 h.

The pH conditions are shown in Figure 6A,B. As the pH increased, the removal rate of 3-keto-DON increased and then decreased. pH 7 and 8 were optimal for AKR4 to degrade 3-keto-DON, with removal rates of 66.28% and 62.55%, respectively (Figure 6A). AKR4 still had more than 80% residual activity after incubation for 12 h at pH 5–9 (Figure 6B).

As shown in Figure 6C, the optimal degradation temperature of AKR4 for 3-keto-DON was 50 °C. The degradation ability of AKR4 for 3-keto-DON decreased rapidly at 70 °C. When the temperature was between 30–50 °C, the removal rate of AKR4 for 3-keto-DON was more than 60%. AKR4 was incubated at various temperatures for 2 h and tested for activity at 30 °C (Figure 6D). It was resistant toward incubation at 35 °C, and about 40% of residual activity was detected after 2 h. A continuous decrease in activity was observed when AKR4 was incubated at 45 °C and 50 °C. AKR4 was stable at 25 °C and 30 °C, and the 3-keto-DON degradation activity was more than 80% of the initial activity after incubation for 2 h.

### 3.8. Degradation Products of 3-Keto-DON

**Figure 7:**
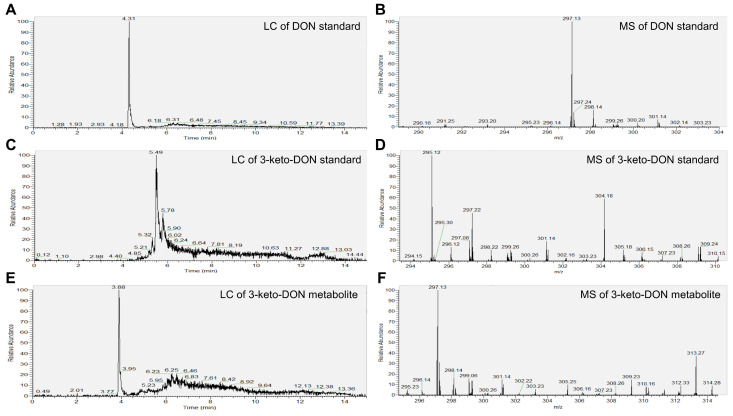
The degradation product of 3-keto-DON. (**A**) Liquid chromatography of DON standard. (**B**) Mass spectrometry of DON standard. (**C**) Liquid chromatography of 3-keto-DON standard. (**D**) Mass spectrometry of 3-keto-DON standard. (**E**) Liquid chromatography of 3-keto-DON metabolite. (**F**) Mass spectrometry of 3-keto-DON metabolite.

As shown in Figure 7D, the 3-keto-DON standard was detected in positive ion mode with a molecular weight of 295.12 [M + H]^+^. The degradation product of 3-keto-DON by AKR4 was detected at 3.88 min (Figure 7E) with a molecular weight of 297.13 [M + H]^+^ in positive ion mode (Figure 7F). Meanwhile, the DON standard was detected at 4.31 min (Figure 7A) with a molecular weight of 297.13 [M + H]^+^ (Figure 7B). It was found that the metabolite of 3-keto-DON had the same molecular weight of 297.13 as DON. However, the retention time of the metabolite of 3-keto-DON was different from that of DON in the same separation program. Therefore, the degradation product of 3-keto-DON by AKR4 was assumed as 3-*epi*-DON, which was the only reported C3-OH epimer of DON among 3-keto-DON degradation products.

### 3.9. Binding Model of 3-Keto-DON to AKR4 by Molecular Docking

**Figure 8:**
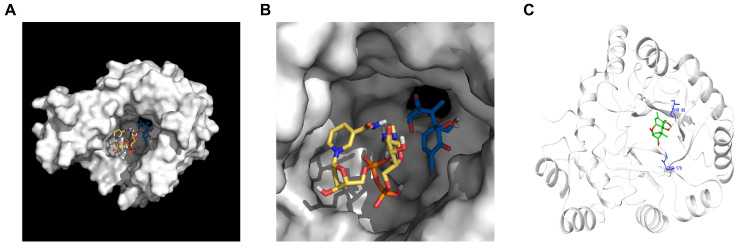
Binding model of 3-keto-DON to AKR4 by molecular docking predicted by AutoDockTools 1.5.6. (**A**) Full view of the binding pattern of 3-keto-DON and AKR4. (**B**) Enlarged view of the details of the binding region of 3-keto-DON to AKR4. The blue structure indicates 3-keto-DON and the yellow structure indicates NADPH. (**C**) The 3D interaction model of 3-keto-DON with AKR4.

The structure of AKR4 was constructed by homology modeling. Then, the interaction of AKR4 and 3-keto-DON was investigated with docking simulations. The most likely docking modes were obtained by the combination of interaction and scoring (Figure 8A). The results indicated that 3-keto-DON and NADPH were located in the same binding pocket and were close to each other (Figure 8B). Hydrogen bonding contributed to the stability of docking (Figure 8C).

## 4. Discussion

Various microorganisms have been reported to degrade DON to 3-*epi*-DON, including *Nocardioides* sp. strain WSN05-2 [28], *Paradevosia shaoguanensis* DDB001 [29], and *Lactobacillus rhamnosus* SHA113 [30], etc. The potential toxicity of bacteria applied to food and feed can be avoided by enzymatic degradation of DON. However, most research has focused on the identification of enzymes that transform DON to 3-keto-DON [20,21,22,25]. Studies have demonstrated that 3-keto-DON and 3-*epi*-DON exhibit low toxicity: the toxicity of 3-keto-DON is 10 times lower than DON, while the toxicity of 3-*epi*-DON is 1181 times lower than DON [18,20,31]. Therefore, there is an urgent need to isolate new enzymes capable of converting 3-keto-DON to 3-*epi*-DON.

Our team previously utilized *P. halotolerans* ANSP101 to degrade DON and found that 3-keto-DON was the resulting degradation product [24]. However, *P. halotolerans*, as a bacterium, can be somewhat limited in its application in food and feed, despite its ability to degrade DON. Therefore, it is necessary to mine the DON-detoxifying enzyme from this strain. It has been reported that DDH from *P. halotolerans* ANSP101 could degrade DON to 3-keto-DON [25]. Recent studies revealed that the first enzyme converting DON to 3-keto-DON and the second enzyme converting 3-keto-DON to 3-*epi*-DON in DON epimerization pathway may exist in a strain at the same time [21,22,23], so there may also be an enzyme in *P. halotolerans* ANSP101 that converts 3-keto-DON to 3-*epi*-DON. In this study, we discovered and characterized a novel enzyme named AKR4 from *P. halotolerans* ANSP101, which was able to degrade 3-keto-DON to 3-*epi*-DON using NADPH as the cofactor. Based on this result, it was speculated that 3-*epi*-DON was not detected during the degradation of DON by *P. halotolerans* ANSP101 possibly because one or some of the metabolites in strain ANSP101 could competitively bind the binding site of 3-keto-DON on AKR4 (Figure 8B), thus inhibiting the degradation of 3-keto-DON by AKR4 after DON was transformed to 3-keto-DON by DDH.

The protein AKR4 from *P. halotolerans* ANSP101 was included in the “aldo/keto reductases” category according to the results of gene annotation. In addition, the NCBI BLAST search results showed that most of the proteins with high homology to AKR4 were aldo/keto reductases. Nearly all of the aldo/keto reductases have a conserved catalytic tetrad containing aspartate, tyrosine, lysine, and histidine [27]. Alignment of the amino acids of AKR4 and aldo/keto reductases obtained by NCBI BLAST search indicated that AKR4 carries a conserved catalytic tetrad across all of aldo/keto reductases (Asp60, Tyr65, Lys89, and His131) (Figure 3). Therefore, it was speculated that AKR4 represents a member of the aldo/keto reductases family. As NAD(P)^+^/NAD(P)H-dependent oxidoreductases, aldo/keto reductases show broad substrate specificity in the detoxification of reactive aldehyde and the degradation of secondary metabolite, etc. [32]. In the present study, the reaction from 3-keto-DON to 3-*epi*-DON was a ketone reduction catalyzed by AKR4.

AKR4 was clearly a NADPH-dependent aldo/keto reductase. When catalyzed using NADH as the cofactor, it was more than 50-fold less efficient over 150 min. This result was consistent with the conversion of 3-keto-DON to 3-*epi*-DON by DepB from *D. mutans* 17-2-E-8 [23] and two enzymes, AKR13B2 and AKR6D1, from *Devosia* strain D6-9 [21] reported previously. It was interesting that an enzyme in *Sphingomonas* sp. strain S3-4, AKR18A1 (ASY03293.1), with 29.97% sequence similarity to AKR4 was capable of transforming DON to 3-keto-DON with NADP^+^ as the cofactor [20]. The interaction between the active sites and the substrate merits further investigation. AKR4 functions at a broad range of pH, from 5 to 10, and broad range of temperature, from 20 to 60 °C. The highest activity of AKR4 degrading 3-keto-DON was at a pH of 7 and a temperature of 50 °C. AKR4 retained more than 70% of the residual activity in the pH range of 4 to 9, and 34% of the residual activity even at pH 3 within 12 h. The high stability of AKR4 under neutral or lower pH will be advantageous for the retention of activity in the gastrointestinal tract of livestock with a pH range from 3 to 7. A 2 h heat treatment at 30 °C had little influence on AKR4 activity, while heat treatment at 40 °C reduced AKR4 activity. However, AKR4 showed a high 3-keto-DON removal rate of 88% at 40 °C, which was suitable for 3-keto-DON degradation in the gastrointestinal tract of livestock. With future study, directed evolution or other approaches may be used to improve the thermostability of AKR4 to make it more suitable for practical applications.

It has been reported that most of the *Devosia* species were able to reduce 3-keto-DON to 3-*epi*-DON and DON simultaneously, except for *D. mutans* 17-2-E-8, which transformed 3-keto-DON to 3-*epi*-DON completely [19]. In the present study, it was confirmed that 3-keto-DON was only converted to 3-*epi*-DON by AKR4 after the reduction of carbonyl group at the C3 position of 3-keto-DON to hydroxyl. As such, this is a positive and effective catalytic process.

In order to understand how AKR4 affects the degradation of 3-keto-DON, we carried out molecular docking studies. The 3-keto-DON was docked into AKR4 in different conformations, and the best docking conformation with a high docking score was selected for subsequent analysis. The docking result showed that there were hydrogen bonds between 3-keto-DON and residues Thr88 or Thr179 of AKR4 (Figure 8C). Amino acid sequences alignment of AKR1, AKR2, AKR3, and AKR4 showed that all of the four enzymes share a conserved catalytic tetrad. However, the amino acids at position 88 and 179 were different (Figure S2). The position 88 of AKR1, AKR2, and AKR4 were Thr, while the corresponding position of AKR3 was Ser. In addition, the amino acid at position 179 of AKR3 and AKR4 were both Thr, while corresponding position of AKR1 and AKR2 were Ala and Cys, respectively. It was speculated that the non-degradation ability of the other three aldo/keto reductases except for AKR4 to 3-keto-DON may be related to the substitution of residues at the two 3-keto-DON binding sites. This result further demonstrated the importance of 3-keto-DON binding residues Thr88 and Thr179 for the 3-keto-DON degradation of AKR4.

## 5. Conclusions

In the present study, the aldo/keto reductase AKR4 from *P. halotolerans* ANSP101 heterologously expressed in *E. coli* BL21 was capable of degrading 3-keto-DON effectively. The optimum reaction conditions for AKR4 to degrade 3-keto-DON were pH 7 and 50 °C. We also confirmed that the degradation product of 3-keto-DON by AKR4 was 3-*epi*-DON, a compound which is essentially non-toxic. Further investigations are required to unveil the combined effect of AKR4 and DDH, the enzyme responsible for oxidation of DON into 3-keto-DON in *P. halotolerans* ANSP101. These results have promising implications for the development of detoxifying enzyme preparations for DON in food and feed industries.

## Data Availability

The original contributions presented in the study are included in the article, further inquiries can be directed to the corresponding author.

## Supplementary Materials

The following supporting information can be downloaded at: https://www.mdpi.com/article/10.3390/foods13071064/s1, Table S1: Primers used in this study; Table S2: Protein sequence comparison between aldo/keto reductases from ANSP101 and DepB; Figure S1: The SDS-PAGE of AKR1, AKR2, AKR3, and AKR4; Figure S2: Multiple sequence alignment of AKR1, AKR2, AKR3, and AKR4 using the PROMALS3D multiple sequence and structure alignment server (prodata.swmed.edu/promals3d/, accessed on 5 October 2023).
