# Supplementary material for: A NADPH-Dependent Aldo/Keto Reductase Is Responsible for Detoxifying 3-Keto-Deoxynivalenol to 3-epi-Deoxynivalenol in Pelagibacterium halotolerans ANSP101
Source: Foods. 2024 Mar 29;13(7):1064. doi: 10.3390/foods13071064 (PMC11011506; doi:10.3390/foods13071064)
Supplement: Supplementary file 1 [file foods-13-01064-s001.zip › foods-2906138-supplementary.pdf]

Supplementary data

Table S1. Primers used in this study.

[a] The underlined sequences indicate restriction enzyme recognition sites.

Gene	Primer	Sequence (5′ to 3′)[a]	Usage
AKR1	*AKR1*-F	TTCCATATGGATTATCGGTCACTTGG	Cloning of
	AKR1-R	CTTCTCGAGGACCATCGGCGGATTG	
AKR2	*AKR2*-F	GGAATTCCATATGACGTTCGGCTCG	Cloning of
	AKR2-R	ATTCTCGAGAACGTGCCCGGCTA	
AKR3	*AKR3*-F	GGAATTCCATATGCAACAGCGCACG	Cloning of
	AKR3-R	ATCATTCTCGAGGGCGAAGGTGCGC	
AKR4	*AKR4*-F	GCTTATTCATATGAGCGATATCAACGCC	Cloning of
	AKR4-R	AATTATTCTCGAGGCTGCGGCCGG	

Table S2. Protein sequence comparison between aldo/keto reductases from ANSP101 and DepB.

Protein	GenBank accession	Number of amino	Identity with
DepB	KFL28068.1	343	100%
AKR1	QJR18724.1	344	44.05%
AKR2	QJR20736.1	303	38.05%
AKR3	QJR17462.1	345	31.62%
AKR4	QJR19719.1	286	31.05%

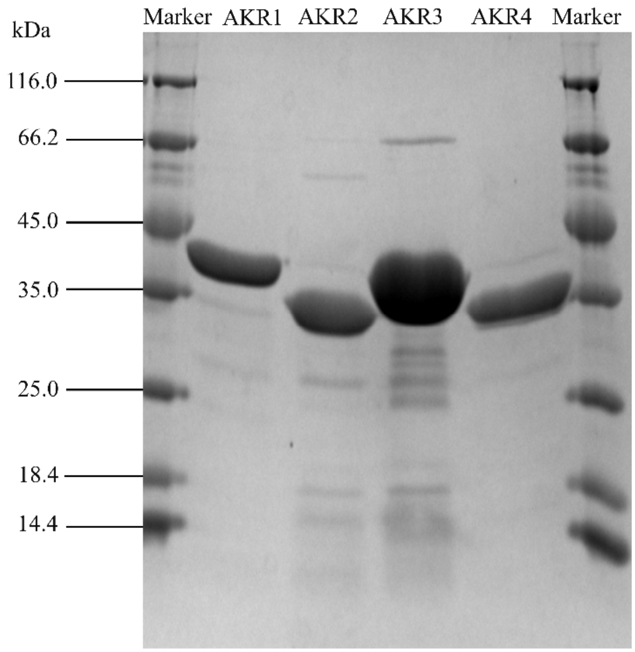

Figure S1. The SDS-PAGE of AKR1, AKR2, AKR3, and AKR4.

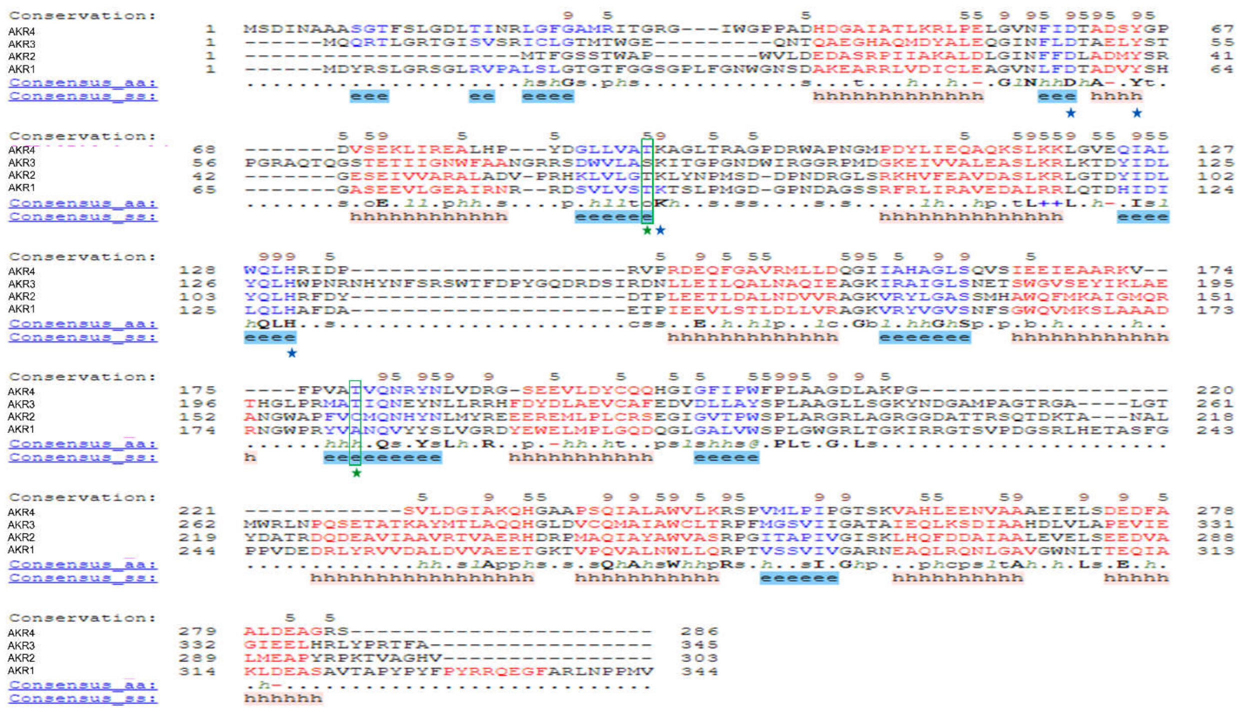

Figure S2. Multiple sequence alignment of AKR1, AKR2, AKR3, and AKR4 using the PROMALS3D multiple sequence and structure alignment server (prodata.swmed.edu/promals3d/). The catalytic tetrad residues were marked with blue stars. The 3-keto-DON binding residues were marked with green stars.
